# Associations between partial foot amputation level, gait parameters, and minimum impairment criteria in para-sport: A research study protocol

**DOI:** 10.1016/j.smhs.2021.11.001

**Published:** 2021-11-12

**Authors:** Fábio C. Lucas de Oliveira, Samuel Williamson, Clare L. Ardern, Neil Heron, Dina Christa Janse van Rensburg, Marleen G.T. Jansen, Sean O'Connor, Linda Schoonmade, Jane Thornton, Babette M. Pluim

**Affiliations:** a Center for Interdisciplinary Research in Rehabilitation and Social Integration, CIUSSS-CN, Université Laval, Quebec City, Quebec, Canada; b Research Unit in Sport and Physical Activity (CIDAF), Faculty of Sport Sciences and Physical Education, University of Coimbra, Coimbra, Portugal; c English Institute of Sport, London, UK; d Division of Physiotherapy, Karolinska Institute, Stockholm, Sweden; e Center for Public Health, Queen's University Belfast, Belfast, UK; f Department of General Practice, Keele University, Staffordshire, UK; g Section Sports Medicine, Faculty of Health Sciences, University of Pretoria, Pretoria, South Africa; h Royal Dutch Lawn Tennis Association (KNLTB), Amstelveen, the Netherlands; i Centre for Human Movement Sciences, University Medical Centre Groningen, University of Groningen, Groningen, the Netherlands; j School of Medicine, Dentistry and Biomedical Sciences Centre for Public Health, Queen's University Belfast, Belfast, UK; k Medical Library, Vrije Universiteit Amsterdam, Amsterdam, the Netherlands; l Department of Family Medicine, Schulich School of Medicine & Dentistry, University of Western Ontario, London, Ontario, Canada; m Department of Epidemiology & Biostatistics, Schulich School of Medicine & Dentistry, University of Western Ontario, London, Ontario, Canada; n AMC/VUmc IOC Research Center of Excellence, Amsterdam Collaboration on Health & Safety in Sports (ACHSS), Amsterdam, Netherlands

**Keywords:** Amputees, Congenital deficiency, Gait analysis, Paralympic sport, Partial foot amputation, Wheelchair tennis, PRISMA, Preferred Reporting Items for Systematic Reviews and Meta-Analysis, PRISMA-ScR, Preferred Reporting Items for Systematic Reviews and Meta-Analysis – Extension for scoping reviews, PECC, population, exposure, concept, and context, MeSH, Medical Subject Headings, PFA, partial foot amputation, MIC, minimum impairment criteria

## Abstract

Altered biomechanics due to amputation can contribute to substantial limitations, influencing sporting activities. Individuals with lower extremity amputations or congenital lower limb deficiency are encouraged to participate in para-sports. However, to compete in Paralympic sports, the candidate must have an impairment that results in lower extremity loss of function and meets or exceeds the sport's minimum impairment criteria (MIC). This review will focus on the MIC for competitive wheelchair tennis. Limb deficiency is known as one of the MIC used to regulate participation in competitive para-sports since it impacts gait, kinematics, and biomechanics of both the upper and lower body. Notwithstanding, it is questionable whether the MIC concerning limb deficiency is set at the correct level for determining eligibility for participating in Paralympic sports. This study aims to provide an overview of the evidence examining the impact of different partial foot amputation (PFA) levels on gait as a proxy for sporting performance. This scoping review will be based on a 6-step methodological framework and Preferred Reporting Items for Systematic Reviews and Meta-Analysis, extension for scoping reviews (PRISMA-ScR). Studies will be selected from PubMed, Embase, CINAHL, and SPORTDiscus. Two authors will screen the titles/abstracts independently. Selected studies will be scrutinised, and the same authors will extract data. Findings will be relevant to informing the evidence-based development of MIC for lower limb impairment after PFA and may be extrapolated to specific Paralympic sports, including wheelchair tennis. Results will be disseminated through scientific publications and conferences to audiences interested in Paralympic sports.

## **What is known about this subject?**

Limb deficiency is currently known as one of the Minimum Impairment Criteria (MIC) used to regulate participation in competitive para-sports since it impacts gait, kinematics, and biomechanics of both the upper and lower body. Notwithstanding, it is questionable whether the MIC concerning limb deficiency is set at the correct level for determining eligibility for participating in Paralympic sports.

## **What are the new findings and/or impact on clinical practice?**

Findings from this scoping review will be relevant to informing the evidence-based development of MIC for lower limb impairment after PFA and may be extrapolated to specific Paralympic sports, including wheelchair tennis.

## Background

Lower extremity amputation is a condition associated with a significant impact on quality of life, morbidity, and mortality.^1^ Chronic degenerative disease complications, including vascular disease due to Type I and Type II diabetes, peripheral vascular disease, and infection, are the most frequent indication for lower-extremity amputation,^2^ with diabetes and associated comorbidities accounting for more than 50% of all lower-limb amputations globally.^3^ In children and young adults, the most common causes include trauma (75%), malignancy (5%), and congenital limb deficiency (5%).^2^ Even though children and young adults have a lower incidence of amputation than older adults, children and young adults contribute significantly to the overall prevalence of lower limb loss due to their longer life expectancy.^3^ One-year mortality rates range from 23% to 53%, depending on the anatomical level of amputation, patient age, and underlying illness.^4^

Altered body biomechanics resulting from an amputation contribute to substantial functional limitations, influencing physical activity levels, participation in sporting activities, and completing activities of daily living.^5^, ^6^, ^7^ The distribution of dysvascular lower leg amputations varies, with the most common level being at the toe (33%), followed by transtibial (28%), transfemoral (26%), and within the foot (11%). Less common levels are ankle disarticulations (Syme), through-knee amputations, hip disarticulation, and hemipelvectomy (combined 1.5%).^1^^,^^4^

### Lower limb deficiency and sport

Individuals with lower extremity amputations or congenital lower limb deficiency are encouraged to participate in recreational and non-competitive para-sports. However, to be eligible to compete in competitive Paralympic sports, the candidate must have an impairment caused by a permanent underlying health condition that results in a substantial loss of function in one or both lower extremities and meets or exceeds the sport's minimum impairment criteria.^8^

The International Paralympic Committee Athlete Classification Code mandates that the International Sports Federations develop sports-specific classification systems.^8^ These systems are mandatory to have an evidence-based focus on the relationship between impairments, essential performance determinants, and athlete input. Ten eligible impairment types in the Paralympic Movement exist: impaired muscle power, impaired passive range of motion, limb deficiency, leg length difference, short stature, hypertonia, ataxia, athetosis, vision impairment, and intellectual impairment. Each impairment type has minimum impairment criteria.

This review will focus on the minimum impairment criteria for a player with a limb deficiency to be eligible to play competitive wheelchair tennis. These criteria are defined as follows: “Complete unilateral amputation of half of the foot length (i.e., measured on the non-amputated foot from the tip of the great toe to the posterior aspect of the calcaneus) or equivalent minimum congenital limb deficiency”.^9^^,^^10^ Since a limb deficiency impacts gait as well as kinematics and biomechanics of both the upper and lower body, investigation as to whether the minimum impairment criteria are set at the correct level for determining eligibility is an important consideration.

Using a scoping review methodology,^11^ this study aims to provide an overview of the evidence examining the impact of different partial foot amputation levels on gait as a proxy for sporting performance.

## Methods and methods

This scoping review will be based on the 6-step methodological framework developed by Arksey and O'Malley^12^ and Levac et al.^13^ The review will be conducted following the Preferred Reporting Items for Systematic Reviews and Meta-Analysis (PRISMA) extension for scoping reviews.^14^ The PRISMA extension for Scoping Reviews (PRISMA-ScR) Checklist will be completed.^11^^,^^14^

### Research question

Our scoping review is designed to answer the following research question: *“What gait parameters are associated with different levels of partial foot amputation?”*

This question was framed based on the elements of the PECC (population, exposure, concept, and context) and is available in Table 1. This will help determine the level of partial foot amputation (including toe, ray, metatarsophalangeal, transmetatarsal, tarsometatarsal, and transtarsal), where the negative effect on gait becomes significant.

**Table 1:** Elements used for determining the research question of this scoping review.

Population	Exposure	Concept	Context
Individuals (aged ≤ 16 years and > 16 years).	Partial foot amputation.	Analysis of gait-related outcomes includes walking speed/velocity, cadence, step length, stride length, step width, stance step duration, peak GRF, center of pressure excursion, and others.	All study designs.

GRF: ground reaction force.

### Search strategy

A comprehensive search will be performed in the following bibliographic databases: PubMed, Embase.com, CINAHL, and SPORTDiscus (via Ebsco). Dates for search are from inception to February 1st^,^ 2021, in collaboration with a medical librarian (LS). Search terms will include controlled terms (Medical Subject Headings [MeSH] in PubMed and Emtree in Embase, Headings in CINAHL, and Thesaurus terms in SPORTDiscus) as well as free text terms. The following terms (including synonyms and closely related words) will be used as index terms or free-text words: ‘amputation’ and ‘forefoot’ or ‘foot joint’ and ‘gait’. The search will be performed without date or language restrictions. Duplicate articles will be excluded. Results from the literature search will be presented in a flowchart following the PRISMA guidelines. The entire search strategies for all databases will be published as Supplementary Information.

Box 1 outlines the preliminary pilot search at the PubMed/Medline conducted in January 2021 to confirm the feasibility of the scoping review and the effectiveness of the search strategy in locating relevant studies related to the topic of interest.

Box 1 **Preliminary pilot search at the PubMed/Medline**

**Table:** 

Search	PubMed Query – February 1, 2021	Results
#4	#1 AND #2 AND #3	315
#3	“Physical Functional Performance"[Mesh] OR “Gait"[Mesh] OR “Gait Analysis"[Mesh] OR “gait∗"[tiab] OR biomechanics[tiab] OR “functional performance"[tiab] OR “functional test∗"[tiab] OR ((motion[tiab] OR movement[tiab] OR moving[tiab] OR locomotion[tiab] OR walk∗[tiab] OR ambulati∗[tiab]) AND analys∗[tiab])	221,983
#2	“Forefoot, Human"[Mesh] OR “Foot Joints"[Mesh] OR “forefoot"[tiab] OR “midfoot"[tiab] OR “toe"[tiab] OR “toes"[tiab] OR “hallux"[tiab] OR “metatars∗"[tiab] OR “intertars∗"[tiab] OR “midtars∗"[tiab] OR “transtars∗"[tiab] OR “intermetatars∗"[tiab] OR “transmetatars∗"[tiab] OR “tarsometatars∗"[tiab] OR “foot joint∗"[tiab] OR “tarsal joint∗"[tiab] OR “ray"[tiab] OR “lisfranc"[tiab] OR “chopart∗"[tiab]	431,368
#1	“Amputation"[Mesh] OR “amputat∗"[tiab] OR “disarticulat∗"[tiab]	51,468

Mesh, Medical Subject Headings. tiab, title and abstract.

Alt-text: Box 1

### Selection and screening of relevant studies

Following the search, studies will be collated and uploaded into Endnote X9.3.3 and duplicates removed. Thereafter, studies will be imported to Rayyan Systems, a free online platform that uses a process of semi-automation for organising systematic reviews^15^ for screening procedures.

The following steps will be followed to identify relevant studies: (1) titles and abstracts will be independently screened by two reviewers, using a blinded standardised protocol; (2) full-text of potentially eligible studies will be independently scrutinised by pairs of reviewers to decide on inclusion; (3) manual searching of the references of the retrieved studies will be conducted to identify additional relevant studies; and, finally, (4) selected studies will be scrutinised for extraction of relevant data.

Disagreements on inclusion will be resolved by consensus between assessors. If required, a third reviewer not involved in the screening will be available to complete final decisions on inclusion.

### Inclusion criteria

This scoping review will include any study focused on gait analysis, or other related outcomes, in individuals who have undergone a partial foot amputation.

No restrictions by year, geographical location, gender, sex, or language will be applied to the search strategies. Relevant studies published in a language that fewer than two assessors are fluent in will be translated to English before its screening. Table 2 contains information describing the characteristics of the included studies in the scoping review. Reasons for exclusion will be reported in the final manuscript and presented in a flowchart following the PRISMA guidelines.^14^

**Table 2:** Criteria for inclusion of studies after the full-text screening.

	Inclusion criteria	Exclusion criteria
**Population**	Individuals (aged ≤ 16 years and > 16 years) who underwent a PFA.	Cadaveric, animals, non-human studies
**Types of PFA**	Partial foot amputation: - (Big) toe - Metatarsophalangeal - Ray amputation - Transmetatarsal - Tarsometatarsal (Lisfranc) - Transtarsal (Chopart)	Level of amputation more proximal than transtarsal (e.g., Pirigoff, Boyd, and Symes) Use of mobility aids such as: - Crutches - Walking stick - Cane - Nordic walking poles
**Outcomes**	- gait speed - cadence - stride length - step length - step width - stance step duration - peak GRF - center of pressure excursion	- stair climbing - self-care
**Study design**	Study design: - peer-reviewed original articles - quantitative, qualitative, mixed, and multimethod design - narrative and systematic reviews - meta-analyses - grey literature	Books, chart reviews, opinion papers, news and magazine articles, study protocols, case reports with less than three cases, dissertation or thesis, editorials, annals of congresses, conference proceedings, presentations, posters
**Study availability**	Full-text available	

GRF: ground reaction force.

### Data charting and extraction

Once full-text screening is completed and studies to be included are identified, two reviewers will independently extract data from five to 10 studies as a pilot exercise. This procedure step is to confirm that our approach follows the research question and purpose of the scoping review.^15^ Eligible studies will be divided among the same two reviewers for completing data extraction.

Data will be inserted in a structured charting table created in a Microsoft Excel spreadsheet, specifically for this scoping review, to summarise the evidence.

Data extracted will comprise the following information: study characteristics (authors, year of publication, country of origin), study population (age, sample size, health status, type of amputation, level of amputation, reason for amputation), objective/purpose, study design (intervention description, duration of the study, number of study arms, measurements, comparators), outcome measures (gait speed, cadence, stride length, step length, step width, stance step duration, peak ground reaction force, center of pressure excursion), main results (related to the outcomes of interest), reported adverse events, and clinical recommendations.

Study authors will be contacted if relevant data for the scoping review are missing or if clarification or additional information is needed.^12^ Divergences that arise between reviewers will be resolved by consensus. Again, a third reviewer will make the final decision when no between-reviewer consensus is achieved.

### Collating, summarizing, and reporting results

This scoping review is commissioned to inform and provide (1) insight on the impact of different partial foot amputation levels on gait and (2) recommendations on the correct minimum impairment criteria for determining sporting eligibility. Findings will also be organised and synthesised for extrapolation to specific Paralympic sports, namely wheelchair tennis.

Results from the scoping review will be clustered into thematic areas and reported in a narrative format with tables and illustrations. A summary of the evidence gathered from the included studies will also be provided in our scoping review.

### Expert consultation

Subject matter experts in gait analysis and amputation topics will be consulted to input specific situations observed throughout the study.

### Ethics and dissemination

Since the methodology of this scoping review comprises reviewing published data, ethics approval will not be needed for this scoping review.

Findings from this review will be disseminated through publications in peer-reviewed journals and presentations at international conferences. Findings will also be communicated to audiences with an interest in Paralympic sports.

## Discussion

Previous literature has demonstrated that the level of gait abnormalities is associated with the level of amputation with more proximal or higher amputation increasing the gait abnormalities.

Limb deficiency is currently known as one of the minimum impairment criteria used to regulate participation in competitive para-sports since deficiency impacts gait, kinematics, and biomechanics of both the upper and lower body. Notwithstanding, it is questionable whether the minimum impairment criteria concerning limb deficiency is set at the correct level for determining eligibility for participating in Paralympic sports.

Findings from this scoping review are relevant by providing evidence-based information to develop minimum impairment criteria for lower limb impairment after a partial foot amputation, and these criteria may be extrapolated to specific Paralympic sports, including wheelchair tennis.

## Availability of data and materials

Data sharing does not apply to this protocol as no datasets were generated or analysed during the current study. Notwithstanding, this protocol is registered at the Open Science Framework Registry, and details can be consulted at https://osf.io/8gh9y.

## Funding

This research received no specific grant from any funding agency in public, commercial or not-for-profit sectors. FCLO received a grant from the Koninklijke Nederlandse Lawn Tennis Bond (KNLTB).

## Authors’ contributions

FCLO, SW, and BP contributed to conception and study design. FCLO and BP drafted the manuscript. LS contributed to the searching strategy. MJ, ML, and CA contributed to the manuscript with critical reviews. All authors read and commented on several versions of this protocol and approved its final version.

## Submission statement

In the name of all authors, the main author declares that this work is original, has not been previously published in any form, and is not under review by any other journal.

## Trial registration number

Open Science Framework Registry (8GH9Y) (https://osf.io/8gh9y).

## Conflict of interest

All the authors confirmed that they do not declare any relevant conflict of interest.
